# Supplementary material for: RNA-guided retargeting of Sleeping Beauty transposition in human cells
Source: eLife. 2020 Mar 6;9:e53868. doi: 10.7554/eLife.53868 (PMC7077980; doi:10.7554/eLife.53868)
Supplement: Supplementary file 1. [file elife-53868-supp1.docx]

| **Oligo name** | **Oligo sequence** |
| --- | --- |
| **PCR primers** | |
| **SBfwd_1** | ACCTGCTGTGGGCGGAGGCCCTAAGATGGGAAAATCAAAAGAAATCAGCCAAGAC |
| **SBfwd_2** | AATCGGCCGGCCAAACTGGGCGGAGGCGCACCTGCTGTGGGCGGAG |
| **SBfwd_3** | AATCACCGGTACCATGGGAAAATCAAAAGAAATCAGCCAAGAC |
| **SBrev_1** | AATCGAATTCTAGTATTTGGTAGCATTGCCTTTAAATTGTTTAACTT |
| **N57rev** | AATCGAATTCTAGCGGTATGACGGCTGCGTG |
| **N57rev_2** | CAGGTGCGCCTCCGCCCAGTTTGCGGTATGACGGCTGCG |
| **N57rev_3** | AATCACCGGTCTTAGGGCCTCCGCCCACAGCAGGTGCGCCTCCGC |
| **pUC3** | CGATTAAGTTGGGTAACGCCAGGG |
| **pUC4** | GCTGGCACGACAGGTTTCCCG |
| **HPRT_fwd** | GTAGTCAGGGTGCAGGTCTC |
| **HPRT_rev** | AGAAGTGTCACCCTAGCCTG |
| **Other oligos** | |
| **Stop_top** | [Phos]CCTGAG |
| **Stop_btm** | [Phos]AATTCTCAGGCCGG |
| **sgAluY-1 top** | [Phos]CACCTCCCAAAGTGCTGGGATTAC |
| **sgAluY-1 bottom** | [Phos]AAACGTAATCCCAGCACTTTGGGA |
| **sgAluY-2 top** | [Phos]CACCGCCTGTAATCCCAGCACTTT |
| **sgAluY-2 bottom** | [Phos]AAACAAAGTGCTGGGATTACAGGC |
| **sgAluY-3 top** | [Phos]CACCTTTTGTATTTTTAGTAGAGA |
| **sgAluY-3 bottom** | [Phos]AAACTCTCTACTAAAAATACAAAA |
| **sgHPRT-0 top** | [Phos]CACCGAAGTAATTCACTTACAGTC |
| **sgHPRT-0 bottom** | [Phos]AAACGACTGTAAGTGAATTACTTC |
| **sgHPRT-1 top** | [Phos]CACCTCTTGCTCGAGATGTGATGA |
| **sgHPRT-1 bottom** | [Phos]AAACTCATCACATCTCGAGCAAGA |
| **sgHPRT-2 top** | [Phos]CACCTAAATTCTTTGCTGACCTGC |
| **sgHPRT-2 bottom** | [Phos]AAACGCAGGTCAGCAAAGAATTTA |
| **sgHPRT-3 top** | [Phos]CACCCTGATAAAATCTACAGTCAT |
| **sgHPRT-3 bottom** | [Phos]AAACATGACTGTAGATTTTATCAG |
| **sgL1-1 top** | [Phos] CACCCGCATATTCTCACTCATAGG |
| **sgL1-1 bottom** | [Phos]AAACCCTATGAGTGAGAATATGCG |
| **sgL1-2 top** | [Phos]CACCGGATTCCTTAGCGGTGTGACTGA |
| **sgL1-2 bottom** | [Phos]AAACAGTCAGTGTGGCGATTCCTTAGG |
| **sgL1-3 top** | [Phos]CACCGTATATACCCAGTAATGGGA |
| **sgL1-3 bottom** | [Phos]AAACTCCCATTACTGGGTATATAC |
| **N57 EMSA top** | TACAGTTGAAGTCGGAAGTTTACATACACTTAAG |
| **N57 EMSA bottom** | CTTAAGTGTATGTAAACTTCCGACTTCAACTGTA |
